# The Chosen ONES: Awards Fund Young Investigators

**DOI:** 10.1289/ehp.115-a24

**Published:** 2007-01

**Authors:** Ernie Hood

A major goal of the 2006 NIEHS Strategic Plan encompasses the institute’s desire to “recruit and train the next generation of environmental health scientists.” To begin to achieve that goal, the NIEHS has unveiled a new annual grants program called the Outstanding New Environmental Scientist (ONES) Award. The five-year grants are designed to identify, encourage, inspire, and support outstanding investigators early in their careers, who have not yet received their first R01 grant. The first ONES grants, totaling $3.6 million, were awarded in September 2006 to eight promising young scientists chosen from more than 70 applicants through a rigorous application, review, and interview process.

The program is the brainchild of NIEHS director David Schwartz, who has been concerned for some time about the loss of promising young scientific talent from the field for lack of support. “As a faculty member at Duke,” he says, “I found that the individuals who were particularly vulnerable in terms of their career development were those at that transitional stage between mentored and independent research, and that many very bright, creative people simply were not supported in ways that enhanced their career development.”

Schwartz says the awards are also intended to help attract innovative young investigators to the NIEHS and the environmental health sciences, as well as to support the institutions that are helping new scientists develop their careers. The program’s long-term impact on the field, in terms of both the science and the scientists, could be significant. “These individuals represent very promising early career trajectories that are likely to have a substantial effect on environmental health sciences, and hopefully will evolve into the leaders in the field in the future,” says Schwartz.

To ease that tricky early-career transition, ONES grantees are encouraged to establish and meet annually with an advisory committee comprising senior experts in their disciplines. According to Pat Mastin, chief of the NIEHS Cellular, Organ, and Systems Pathobiology Branch, who helped coordinate the initial ONES process, the grants represent a hybrid between mentored career development awards and independent R01 grants. “We think the young investigators should continue to be mentored,” Mastin says. “So we encouraged them to identify not a specific mentor, but an advisory committee, to give not only scientific advice but also career path advice.”

The grantees recognize and appreciate the value of this hybrid approach to mentoring. “It gives us access to people we wouldn’t normally be interacting with,” says ONES grantee Thomas Begley, an assistant professor in the Department of Biomedical Sciences at the University at Albany State University of New York. “Having a mechanism to ensure that will promote good science on my end, and also will help me network with others in the field.”

Grantee Patricia Opresko, an assistant professor in the Department of Environmental and Occupational Health at the University of Pittsburgh, agrees. “The grant has funds that will allow the four investigators on my advisory committee to come to Pittsburgh and meet with me once a year to focus on my project and offer their ideas, insight, input, and criticisms,” she says. “It adds an additional layer of mentoring that is really critical for a young investigator’s development.”

## Investing in the Future

Funding included in the grants is front-loaded with additional monies in the early years. This allows the investigators to expand and accelerate their research programs by purchasing equipment, adding personnel, and participating in specialized training activities and course work. For each of the five years, the researcher can receive up to $250,000 in direct costs. An additional $150,000 per year is provided in the first two years, with $25,000 per year added in years three through five.

Grantees are expected to devote up to 80% of their time to their research projects in the first two years. This scenario is designed to encourage the scientists to eventually pursue other grant opportunities, gradually becoming more entrepreneurial, more collaborative, and more independent principal investigators.

Not surprisingly, the grantees expect the protected time and generous funding to benefit their research agendas and careers tremendously. “This award allows me to get some equipment into the lab that I would have had trouble getting on my own, and to set up what I term ‘mini-sabbaticals,’ where I can go and seek the additional training that I need to succeed,” says Stephania Cormier, an assistant professor of biological sciences at Louisiana State University.

“Being a physician-scientist, this will give me protected time to focus on research, as opposed to clinical work,” says Gökhan Mutlu, an assistant professor in the Division of Pulmonary and Critical Care Medicine at Northwestern University.

Michael Borchers, a research assistant professor in the Department of Internal Medicine at the University of Cincinnati, is excited that the grant will allow him to expand the scope of his study. “This is going to take me from being a hammer to a jackhammer in terms of trying to bust this research question up,” he says. “The equipment I’m getting is going to add to the validity of my conclusions and the strength and speed with which I can attack the question.”

Donna Zhang, an assistant professor in the Department of Pharmacology and Toxicology at the University of Arizona, sees both short- and long-term rewards. “With this grant, I will be able to do benchtop research, plus I’m going to hire more graduate students and postdocs, so it will definitely move the research faster, to get publication faster and to test the hypothesis faster,” she says. “And I think it will speed up my tenure and my growth in academics.”

Each of the eight inaugural ONES awardees is poised to make significant contributions to knowledge in the environmental health sciences, with potential therapeutic, preventive, or policy translational applications. Their timelines may vary considerably, but all are confident that the ONES grant will accelerate the progress of their research and the realization of actionable results.

## ONES Research Thumbnails

Begley is exploring cell signaling pathways that contribute to DNA repair when damage results from exposures to environmental toxicants. His group has identified a pathway that may govern the production of damage response proteins controlled by multiple tRNA methyltransferases. They will use yeast, human, and mouse cell lines as models to explore the activity of the pathway in response to environmental insults.

Ultimately, the aim is to determine how RNA-based signals coordinate DNA damage response pathways. “Understanding all of the different cellular components that work in response to exposures would be important for predicting individual susceptibility to environmentally induced diseases,” says Begley. “Also, if we can have a better understanding of stress response pathways, they have the potential to be exploited for clinical purposes.”

The ONES award will allow Michelle Bell, an assistant professor of environmental health at the Yale School of Forestry & Environmental Studies, to substantially expand her investigation of the mortality and morbidity effects of tropospheric ozone. With a background in both environmental engineering and epidemiology, she is the sole ONES grantee pursuing an epidemiological project. She plans to use the funding to add to her staff and computer equipment, as well as to take courses and establish collaborations.

“Instead of just looking at whether there’s a link between ozone and mortality, I will investigate by how far deaths are advanced from ozone, which is a very different public health question if it’s just a few days versus . . . a few months or even years,” says Bell. She will also investigate the effects of exposure to complex mixtures of air pollutants, which more closely mirrors the actual environment in which people are simultaneously exposed to many different agents. “I think this work will be very interesting to policy makers, because it’s trying to look at situations in the real world,” she says. “My work is population epidemiology–based; it’s not hypothetical.”

Borchers’ work involves exposures to air pollutants that activate the innate immune system, releasing pulmonary lymphocytes via some of the same mechanisms that release them when the lung is exposed to biological pathogens. Although the molecular pathway involved is apparently conserved, it is presently unclear why the system responds the way it does to toxicants. Borchers is investigating the potential role of this phenomenon in chronic obstructive pulmonary disease (COPD), looking at a pathway that involves NKG2D receptor activation in pulmonary epithelial cells.

“If [the receptor] is activated by a toxicant, it’s unnecessarily causing the immune system to do something disruptive,” he explains. “This hyperresponse may contribute to the pathophysiology of chronic airway diseases, especially in genetically susceptible populations.”

Borchers says the genes he is studying relative to this phenomenon are highly polymorphic. “I want to study the genetic variability of these pathways, to see whether that variability contributes to susceptibility to COPD,” he says. “Out of one hundred people who have a hyperactive immune system, how many of them are hyperreactive due to the polymorphisms in genes associated with activation of the immune system? If you identify a subpopulation, then perhaps you could have directed therapeutics.”

Cormier is seeking to determine whether exposure during early neonatal life to ultrafine particulate matter (PM_0.1_) leads to predisposition to, development of, or exacerbation of allergic respiratory disease in adults. PM_0.1_ is typically produced from incineration of hazardous wastes. As she explains the concept, incineration sites filter PM_2.5_ and larger particles, and there are regulatory restraints on how much PM_2.5_ can be in the air. But research has shown that up to 70% of PM_2.5_ is composed of PM_0.1_ and smaller, and the EPA does not monitor or regulate these ultrafine particles.

PM_2.5_ has been implicated in a variety of adverse health effects; there are spikes in cardiovascular and respiratory events in areas where PM_2.5_ levels exceed EPA standards. There is some suspicion that because the majority of these particles are ultrafine, they may be causing the acute health effects seen in emergency rooms. By studying acute exposures in neonates, Cormier hopes to shed light on the potential physiologic underpinnings of exposure-related adverse events.

She is also looking at how ultrafine particles affect the still-developing lung to determine whether an acute exposure in the neonate predisposes the child to develop a chronic disease state later in life. “Two things could be happening,” says Cormier. “The ultrafine particles could be changing structure—and we have some evidence that the exposure is actually changing genes that control lung structure—or the particles could be changing the immune response, so that you are more prone to an allergic-type response.”

Cormier has previously studied these issues from an infectious disease point of view, looking at how respiratory syncytial virus exposure in neonatal animals permanently changes the lung and the immune system. In her ONES work, she will be using some of these same techniques to see if oxidative stress produced by pollutant particles causes these long-term changes. “The outcome of these studies could have important implications for both public health and environmental policy,” she says.

Sven-Eric Jordt, an assistant professor in the Department of Pharmacology at the Yale University School of Medicine, comes to environmental health science from the pain research field. For his ONES project, he will study the function of sensory neurons in the periphery of the body related to exposure to environmental chemical irritants. This will build upon his previous work on the effects of noxious natural products on sensory neurons and their receptors.

Jordt has found that some of those receptors are also activated by environmental irritants, and that the response is likely to involve the ion channel TRPA1, which is expressed exclusively in sensory fibers that are also sensitive to capsaicin, an irritant derived from chili peppers. “We will try to identify novel receptors for environmental irritants, how these irritants interact with the receptors, and how they are activated and regulated, especially in the background of inflammatory disease and asthma, chronic cough, or allergic conditions,” he says.

Jordt adds that although the role of the immune system in hypersensitivity responses has been well studied in the environmental health sciences, sensory neuroscience is an aspect of the human response to environmental toxicants that has been mostly neglected. He points out that the receptors involved have been intensely studied in the pain research and pharmaceutical arenas to help develop anti-inflammatory agents and analgesics. “We imagine that some of these agents may also reduce irritation by chemical irritants, or reduce hypersensitivity in patients with allergy or asthma, for example,” says Jordt. “So we can basically translate some of the results coming from pain pharmacology or irritant pharmacology into clinical applications in the environmental health sciences.”

Mutlu’s research interest is in how the responses of the alveolar epithelium to air pollution can lead to adverse cardiovascular effects. He hypothesizes that pulmonary exposure to ambient PM may cause the blood to thicken, leading to thrombosis, or clot formation, and that this may be one of the mechanisms to explain how air pollution contributes to heart attacks and strokes. His ONES project will elucidate the physiological mechanisms involved in the hypercoagulation response to PM exposure.

Mutlu’s previous experiments have suggested that this response may involve increased production of the inflammatory mediator interleukin-6 (IL-6). In knockout mice lacking IL-6, exposure to PM did not elicit hypercoagulation. “We can also block it by pretreating the animals with beta blockers, which are well known and commonly used medications for heart disease,” says Mutlu.

Although the phenomenon does not cause disease, Mutlu explains that it accentuates risk of a cardiovascular event in an individual with underlying heart disease or tendency toward stroke. “We will look at the applicability of our findings to human disease,” he says. “If we can confirm our findings, the use of beta blockers may prevent some of the cardiovascular mortality associated with particulate matter.”

Opresko is investigating the mechanisms of genomic instability associated with aging and aging-related diseases, particularly what happens to the telomere structures at the end of the chromosome, which in most human somatic cells dwindle with age. Epidemiological studies have shown that oxidative stress associated with inflammatory diseases, obesity, and smoking can also contribute to an accelerated rate of telomere loss. On a DNA sequence level, the sequences that occur at telomeric ends are rich in guanine runs, and are particularly prone to oxidation.

Environmental metals are an important source of oxidative stress, and Opresko plans to explore the association between exposure to metals and accelerated telomere shortening. “We’re particularly interested in hexavalent chromium,” she says. “This metal is an environmental carcinogen that causes respiratory cancers and respiratory disease due to its route of exposure, and is an occupational hazard associated with the welding industry.”

Opresko explains that hexavalent chromium has been found to induce different types of DNA adducts, chromium adducts, cross-linked adducts, single-strand breaks, and double-strand breaks, particularly in guanine runs. So in addition to causing oxidative stress, it is likely that hexavalent chromium also induces direct DNA damage in these sequences. “We think that hexavalent chromium is an excellent agent and probe to determine the consequences of environmental DNA damage on telomeric integrity, and also to help us understand cellular pathways that repair telomeric damage,” says Opresko. “By understanding these mechanisms, we might be able to design intervention therapies that can help preserve our telomeric ends, and delay the onset of some of the aging-related diseases that are associated with telomere dysfunction.” Such diseases include cancer, macular degeneration, and osteoporosis.

Zhang is studying a chemoprotective cellular pathway called the antioxidant response element–Nrf2–Keap1 signaling pathway, which can be activated by dietary compounds. She hypothesizes that activation of the pathway acts as an endogenous protective system against arsenic-induced toxicity and carcinogenicity.

Drinking water contaminated with arsenic, a known carcinogen, is a worldwide public health problem. However, arsenic is not a mutagen, but induces malignant transformation possibly through an epigenetic or cell signaling mechanism, causing cellular damage through generation of reactive oxygen species. “By understanding the pathway, we can boost people’s antioxidant responses, which could have a broad impact on public health worldwide,” says Zhang.

The pathway itself is mainly controlled by the transcription factor Nrf2. Recent findings have shown that Nrf2 knockout mice have displayed increased sensitivity to chemical toxicants and carcinogens, and are resistant to the protective actions of chemoprotective compounds. In her ONES project, Zhang will seek to elucidate the mechanism of Nrf2 activation to protect against arsenic-induced toxicity and tumorigenicity.

Zhang’s work could also lead to the design of a small molecule pharmaceutical that would up-regulate the antioxidant pathway. “But,” she says, “for people in developing countries where arsenic in drinking water is such a problem and where a pharmaceutical drug is too expensive for them, it will be much cheaper and more readily available if we identify naturally occurring protective compounds in vegetables or plants.” In short, she advises, “Eat your broccoli!”

## Figures and Tables

**Figure:**
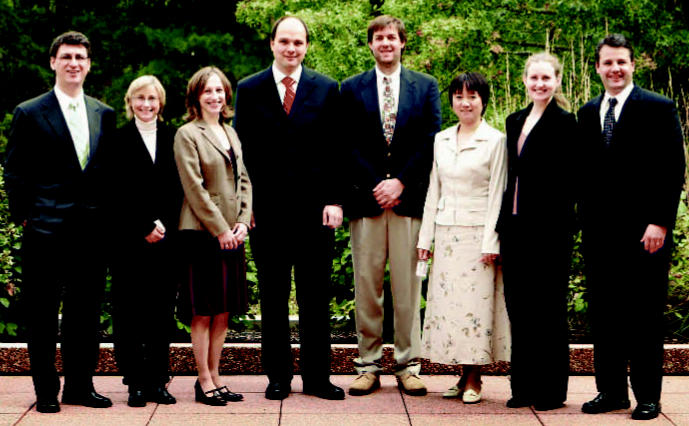
ONES awardees. (left to right) Gökhan Mutlu, Stephania Cormier, Patricia Opresko, Sven-Eric Jordt, Michael Borchers, Donna Zhang, Michelle Bell, Thomas Begley

